# A new species of *Ischnothyreus* Simon, 1893 (Araneae, Oonopidae) from Guangdong Province, China

**DOI:** 10.3897/BDJ.11.e105283

**Published:** 2023-06-02

**Authors:** Hongjin Fu, Zengxue Wang, Yufei Sun, Yanfeng Tong, Dongju Bian

**Affiliations:** 1 College of Life Science, Shenyang Normal University, Shenyang 110034, China College of Life Science, Shenyang Normal University Shenyang 110034 China; 2 Key Laboratory of Forest Ecology and Management, Institute of Applied Ecology, Chinese Academy of Sciences, Shenyang 110016, China Key Laboratory of Forest Ecology and Management, Institute of Applied Ecology, Chinese Academy of Sciences Shenyang 110016 China

**Keywords:** Asia, goblin spider, morphology, Oonopinae, taxonomy

## Abstract

**Background:**

*Ischnothyreus* Simon, 1893 is one of the most speciose genera of Oonopidae, with 124 extant species mainly distributed in the Old World. Currently, 27 species are known in China.

**New information:**

A new species, *Ischnothyreusruyuanensis* Tong, sp. n., is described from Guangdong Province, China. Morphological description and illustrations are provided.

## Introduction

With currently 1891 described species in 115 genera, goblin spiders (Oonopidae) are a diverse spider family (WSC 2023). They are small (0.5–4.0 mm), haplogyne, usually six-eyed spiders and are most diverse in the tropical and subtropical regions (Jocqué and Dippenaar-Schoeman 2006).

The genus *Ischnothyreus* of China have been poorly studied for a long period time. Lee (1966) reported a new recorded species and Brignoli (1974) described one new species from Taiwan. Yin and Wang (1984) described one new species from Yuelu Mountain, Hunan; Xu (1989) described one new species from Anhui; and Hu (2001) described one new species from Tibet. Recently, a series of publications greatly increased the knowledge of the diversity of *Ischnothyreus*. Eight new species from Hainan (Tong and Li 2008, Tong and Li 2012), one new species from Taiwan (Tong and Li 2014), one new species from Chongqing (Tong et al. 2018), one new species from Jiangxi (Liu et al. 2019), seven new species and one newly-recorded species from Yunnan (Huang et al. 2021, Tong et al. 2021) and three new species from Tibet (Tong et al. 2023) have recently been described from China. Up to now, 27 species of *Ischnothyreus* have been recorded in China.

In this paper, a new species of *Ischnothyreus* collected from the leaf litter in Guangdong Province of China is described and illustrated.

## Materials and methods

All the specimens were collected by sifting leaf litter. The specimens were examined using a Leica M205C stereomicroscope. Details were studied under an Olympus BX51 compound microscope. Photos were made with a Canon EOS 750D zoom digital camera (18 mega pixels) mounted on an Olympus BX51 compound microscope. Vulvae were cleared in lactic acid. Male palps and chelicerae were mounted in Kaiser’s glycerol gelatine. Scanning electron microscope images (SEM) were taken under high vacuum with a Hitachi TM3030 after critical-point drying and gold-palladium coating. All measurements were taken using an Olympus BX51 compound microscope and are in millimetres.

All specimens are preserved in 75% ethanol. The type material is deposited in the College of Life Science, Shenyang Normal University (SYNU) in Liaoning, China.

## Taxon treatments

### Ischnothyreus ruyuanensis

Tong sp. n.

9A5E3EE4-9749-5471-B6CA-9D1EBDE27C82

751B67C9-D55C-4763-919D-ED32EFF94219

#### Materials

- **Type status:** Holotype. **Occurrence:** catalogNumber: SYNU-674; recordedBy: Weihua Cheng; individualCount: 1; sex: male; lifeStage: adult; occurrenceID: EEC9560A-524B-5DB2-ADF0-C5871DFD4D51; **Taxon:** scientificName: *Ischnothyreusruyuanensis*; order: Araneae; family: Oonopidae; genus: Ischnothyreus; **Location:** country: China; stateProvince: Guangdong; county: Shaoguan City; locality: Ruyuan Yao Autonomous County, LalingYaozhai; verbatimElevation: 350 m; verbatimCoordinates: 24°45.816'N, 113°14.183'E; **Identification:** identifiedBy: Yanfeng Tong; **Event:** samplingProtocol: sifting leaf litter; eventDate: 04/12/2021
- **Type status:** Paratype. **Occurrence:** catalogNumber: SYNU-675-676; recordedBy: Weihua Cheng; individualCount: 2; sex: female; lifeStage: adult; occurrenceID: C0EF0169-B026-531F-9AE1-EEABBCEF2814; **Taxon:** scientificName: *Ischnothyreusruyuanensis*; order: Araneae; family: Oonopidae; genus: Ischnothyreus; **Location:** country: China; stateProvince: Guangdong; county: Shaoguan City; locality: Ruyuan Yao Autonomous County, LalingYaozhai; verbatimElevation: 350 m; verbatimCoordinates: 24°45.816'N, 113°14.183'E; **Identification:** identifiedBy: Yanfeng Tong; **Event:** samplingProtocol: sifting leaf litter; eventDate: 04/12/2021

#### Description

**Figure 1.**
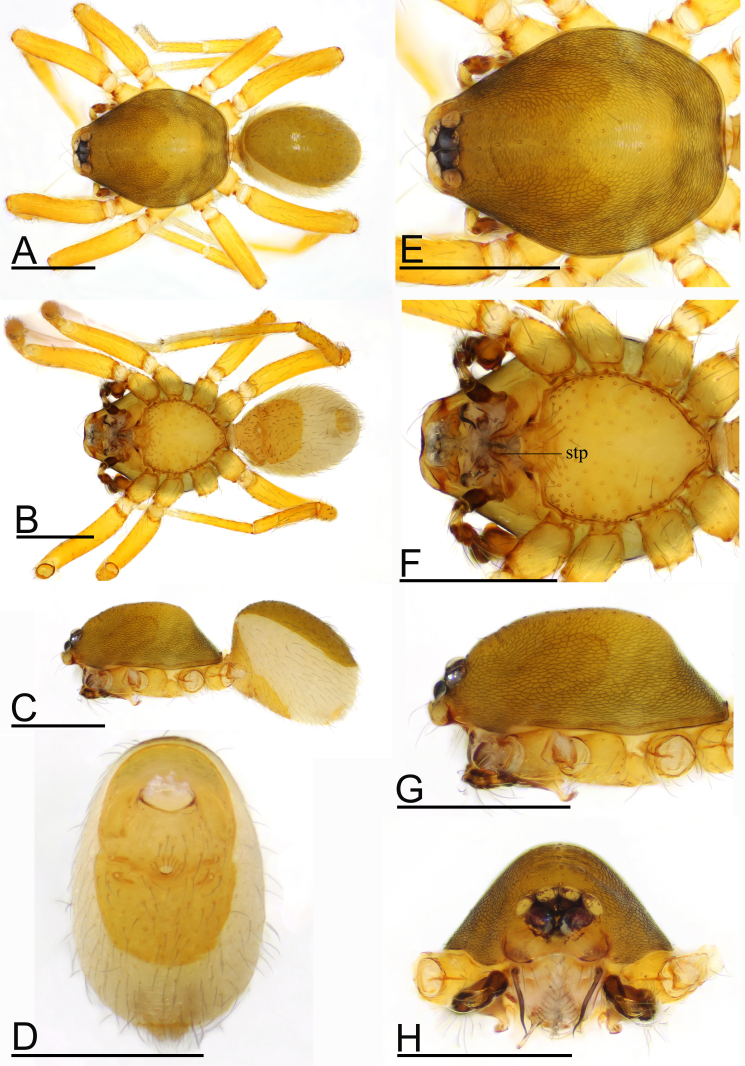
*Ischnothyreusruyuanensis* sp. n., holotype male. **A** habitus, dorsal view; **B** habitus, ventral view; **C** habitus, lateral view; **D** abdomen, ventral view; **E** prosoma, dorsal view; **F** prosoma, ventral view; **G** prosoma, lateral view; **H** prosoma, anterior view. Abbreviation: stp = strong, tooth-like projection. Scales: 0.4 mm.

Male (Holotype). Body: habitus as in Fig. 1A–C; body length 1.37. Carapace: 0.65 long, 0.58 wide; yellow, oval in dorsal view, pars cephalica strongly elevated in lateral view, surface of elevated portion of pars cephalica and sides finely reticulated, lateral margin straight, smooth (Fig. 1E). Clypeus: curved in frontal view, ALE separated from edge of carapace by 0.7 times their diameter (Fig. 1H). Eyes: ALE largest, ALE circular, PME squared, PLE oval; posterior eye row recurved from above; ALE separated by less than their radius, ALE and PLE touching (Fig. 1E and H). Sternum: as long as wide, pale orange (Fig. 1B and F). Mouthparts: chelicerae, endites and labium yellow; chelicerae straight, anterior face with strong, thorn-like process (tlp), base of fangs with very long sclerotised process (lsp), fang groove with a few small denticles (Fig. 3A, G and H); anteromedian tip of endites with one strong, tooth-like projection (stp) (Fig. 1F). Abdomen: 0.64 long, 0.41 wide; dorsal scutum dark brown, covering 4/5 of abdomen width and approximately 5/6 of abdomen length, not fused to epigastric scutum; postepigastric scutum covering 2/3 of abdomen length. Legs: pale orange, femur I with 2 prolateral spines, tibia I with 4 pairs, metatarsus I with 2 pairs of long ventral spines. Leg II spination similar to leg I, except femur with only 1 prolateral spine. Legs III and IV spineless. Palp: trochanter with ventral projection (vp); bulb with two ventral protuberances (vpr), distal end of bulb with dorsal membrane (dm) and large ventral lobe (vl) (Fig. 3B–F).

**Figure 2.**
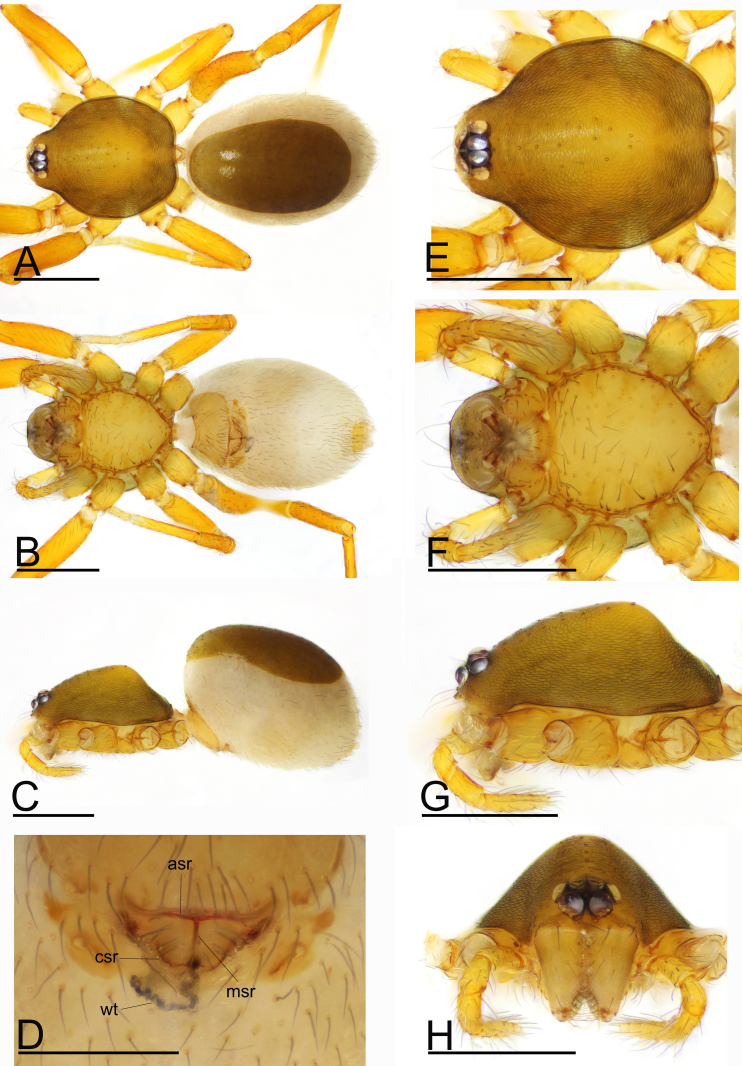
*Ischnothyreusruyuanensis* sp. n., paratype female. **A** habitus, dorsal view; **B** habitus, ventral view; **C** habitus, lateral view; **D** epigastric region, ventral view; **E** prosoma, dorsal view; **F** prosoma, ventral view; **G** prosoma, lateral view; **H** prosoma, anterior view. Abbreviations: asr = anterior sclerotised ridge, csr = curved sclerotised ridge, msr = middle sclerotised ridge, wt = winding tube. Scales: A–C、E–H = 0.4 mm; D = 0.2 mm.

**Figure 3.**
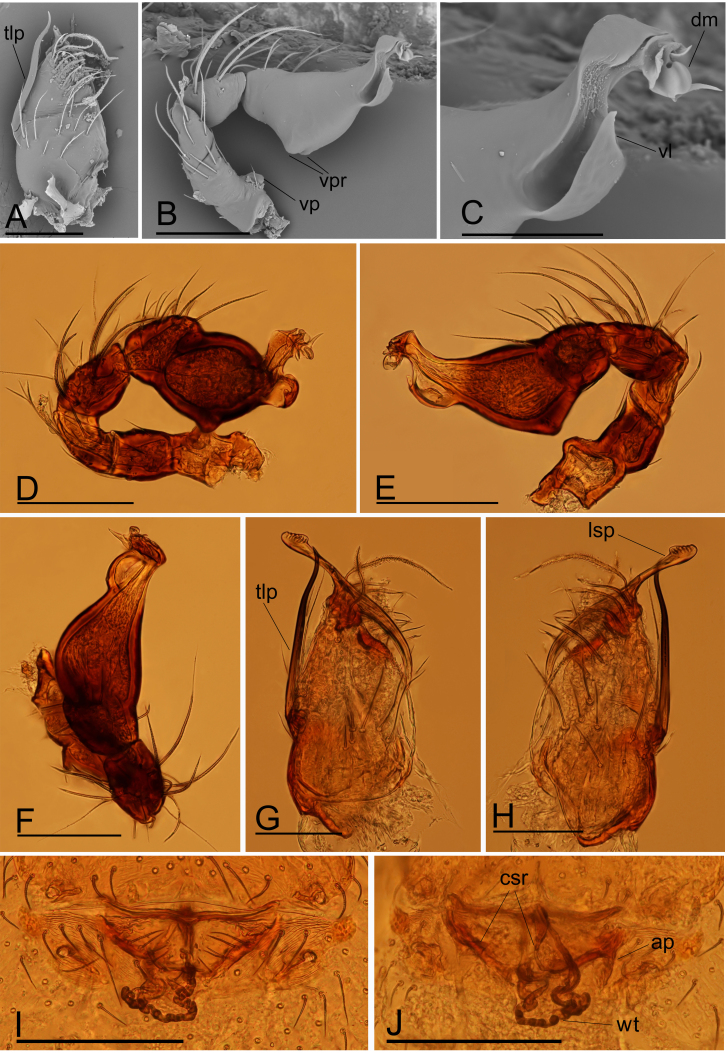
*Ischnothyreusruyuanensis* sp. n., male holotype, A–C (SEM) and D–H (light); female paratype, I–J (light). **A** left chelicerae, anterior view; **B** left palp, prolateral view; **C** distal part of palpal bulb, prolateral view; **D** left palp, prolateral view; **E** left palp, retrolateral view; **F** left palp, dorsal view; **G** left chelicerae, anterior view; **H** left chelicerae, posterior view; **I** endogyne, ventral view; **J** endogyne, dorsal view. Abbreviations: ap = apodemes, csr = curved sclerotised ridge, dm = dorsal membrane, lsp = long sclerotised process, tlp = thorn-like process, vl = ventral lobe, vp = ventral projection, vpr = ventral protuberances, wt = winding tube. Scales: A, B, D–H = 0.1 mm; C = 0.05 mm; I, J = 0.2 mm.

Female (SYNU-675). Same as male, except as noted. Body: habitus as in Fig. 2A–C; body length 1.65. Carapace: 0.61 long, 0.60 wide. Mouthparts: chelicerae and endites unmodified. Abdomen: 0.89 long, 0.62 wide. Epigastric area (Fig. 2D): postepigastric scutum narrow (lenght/width = 3.3), its anterior margin slightly thickened (asr), with dark sclerotised ridge in the middle (msr) of postepigastric scutum. Endogyne (Fig. 3I and J): with 2 strongly curved sclerites (csr), winding tube (wt) long, strongly convoluted.

#### Diagnosis

The new species is similar to *I.spineus* Tong & Li, 2012 in the thorn-like process on the male chelicerae, but can be distinguished by the thorn-like process nearly straight (Fig. 1H, Fig. 3G and H) vs. strongly curved (see Tong and Li (2012): fig. 3D and H), the sclerotised process of fang base more than 1/3 the whole fangs' length (Fig. 3G and H) vs. less than 1/4 the whole fangs' length (see Tong and Li (2012): figs. 3H and 5C) and the dark sclerotised ridge (msr) in the middle of the postepigastric scutum (Fig. 2D) vs. a semi-circular depression (see Tong and Li (2012): figs. 4G and 5D).

#### Etymology

The specific epithet is an adjective referring to the type locality.

#### Distribution

Known only from the type locality.

## Supplementary Material

XML Treatment for Ischnothyreus ruyuanensis
